# Deoxynivalenol Impairs Weight Gain and Affects Markers of Gut Health after Low-Dose, Short-Term Exposure of Growing Pigs

**DOI:** 10.3390/toxins7062071

**Published:** 2015-06-09

**Authors:** Arash Alizadeh, Saskia Braber, Peyman Akbari, Johan Garssen, Johanna Fink-Gremmels

**Affiliations:** 1 Division of Veterinary Pharmacy, Pharmacology and Toxicology, Utrecht University, Yalelaan 104, 3584-CM Utrecht, The Netherlands; E-Mails: a.alizadeh@uu.nl (A.A.); a.peyman@uu.nl (P.A.); j.fink@uu.nl (J.F.-G.); 2 Division of Pharmacology, Utrecht Institute for Pharmaceutical Sciences, Faculty of Science, Utrecht University, Universiteitsweg 99, 3584-CG Utrecht, The Netherlands; E-Mail: j.garssen@uu.nl; 3 Department Immunology, Nutricia Research, Uppsalalaan 12, 3584-CT Utrecht, The Netherlands

**Keywords:** deoxynivalenol, intestine, weight gain, histomorphology, tight junctions, cytokines

## Abstract

Deoxynivalenol (DON) is one of the major mycotoxins produced by Fusarium fungi, and exposure to this mycotoxin requires an assessment of the potential adverse effects, even at low toxin levels. The aim of this study was to investigate the effects of a short-term, low-dose DON exposure on various gut health parameters in pigs. Piglets received a commercial feed or the same feed contaminated with DON (0.9 mg/kg feed) for 10 days, and two hours after a DON bolus (0.28 mg/kg BW), weight gain was determined and samples of different segments of the intestine were collected. Even the selected low dose of DON in the diet negatively affected weight gain and induced histomorphological alterations in the duodenum and jejunum. The mRNA expression of different tight junction (TJ) proteins, especially occludin, of inflammatory markers, like interleukin-1 beta and interleukin-10 and the oxidative stress marker heme-oxigenase1, were affected along the intestine by low levels of DON in the diet. Taken together, our results indicate that even after low-level exposure to DON, which has been generally considered as acceptable in animal feeds, clinically-relevant changes are measurable in markers of gut health and integrity.

## 1. Introduction

Deoxynivalenol (DON, vomitoxin) is the most frequently-occurring type B trichothecene produced by several field fungi, including *Fusarium graminearum* and *Fusarium culmorum,* and is commonly found in cereals and grains, particularly wheat, barley and maize, in areas with a moderate climate. The effects of DON exposure on different animal species related to the concentration and duration of exposure has been described in several reviews [1,2,3,4]. As DON is heat-stable, it resists common processing procedures during feed manufacturing [5]. Exposure to DON can induce gastro-intestinal inflammation and necrosis within the intestinal tract and disturbs the gut barrier function [3,6,7]. In addition, DON is able to cause alterations in brain functions [8,9], induces vomiting (hence the name) and negatively affects growth hormone production [10]. *In vivo* animal studies and practical data support the hypothesis that moderate levels of exposure to DON result in temporary feed refusal, lower feed intake accompanied with a reduction in weight gain, whereas at higher doses of DON, clinical signs of intoxication include emesis, leukocytosis, hemorrhage and even circulatory shock [4,9,11]. *In vivo* and *in vitro* studies present evidence that DON exerts an array of effects at the cellular level, including an increase in pro-inflammatory gene expression [12,13], impairment of cell division, proliferation, differentiation and cell membrane integrity, as well as induction of apoptosis [7,14]. There are obvious species variations in the susceptibility to DON, and pigs show the highest sensitivity to DON. This is in agreement with the high oral bioavailability of DON in pigs, which precedes the detoxification of DON by the flora of the large intestines to de-epoxy DON (DOM), which is less toxic [4,15]. The major concerns related to low-dose exposure to DON are its direct effects on the intestinal barrier and the intestinal immune system, as they may have a significant impact on pig health and performance [3,16,17], as also indicated in the summary of recent *in vivo* piglet studies, as presented in Table 1. The recommended maximum acceptable level for DON according to European Commission Recommendation 2006/576/EC is 0.9 mg/kg feed. Considering the available literature and the EU recommendation, the current study focused on DON-associated effects in the intestinal tract of growing pigs with the aim to identify sub-clinical alterations that might impair animal performance and, hence, serve as biomarkers of low-dose exposure to DON. To this end, pigs were given DON (0.9 mg/kg feed) in the diet for a period of 10 days, whereafter various gut health parameters were investigated.

**Table 1:** Summary of the *in vivo* pig studies related to the effects of dietary deoxynivalenol (DON) on the intestine.

DON	Trial period	Biomarker	DON effects	Reference
4 mg/kg feed	37 days	Oxidative stress markers in blood (catalase (CAT), total antioxidant capacity (T-AOC), hydrogen peroxide (H_2_O_2_), nitric oxide (NO), maleic dialdehyde (MDA) and diamine oxidase (DAO)), kidney, liver and small intestine (H_2_O_2_, MDA and DAO) Intestinal morphology	DON induced oxidative stress DON increased intestinal permeability DON inhibited protein synthesis and cell proliferation	[18]
2.9 mg/kg feed	1 week	DON transport study	Dietary DON affected the jejunal transport of DON	[19]
3.1 mg/kg feed	37 days	Crypt depth Intestinal cell proliferation Immunofluorescence staining zona occludens protein-1 (ZO-1) and β-catenin	No effect on crypt depth No effect on epithelial cell proliferation No effect on apical junction proteins	[20]
2.3 mg/kg feed	35 days	Intestinal morphology/histological score jejunum Mitogen activated protein kinases (MAPK) expression in jejunum	DON induced histological lesions DON activated MAPK extracellular-signal-regulated kinases 1/2 (ERK1/2) and p38	[21]
2.2–2.9 mg/kg feed	11 weeks	Composition and perforation of the basement membrane of intestinal villi Presence of CD16^+^ cells or their dendrites in the epithelium	DON increased the pore number in jejunum DON increased the number of CD16^+^ cells in the epithelium of the jejunum	[22]
4 mg/kg feed	30 days	Intestinal morphology Intestinal function	DON enhanced intestinal permeability, damaged villi, caused epithelial cell apoptosis and inhibited protein synthesis	[23]
3 mg/kg feed	5 weeks	Intestinal morphology, intestinal cytokine expression Tight and adherens junction protein expression (occludin (OCLN), E-cadherin)	DON induced atrophy and fusion of villi DON decreased villi height and cell proliferation in the jejunum DON reduced number of goblet cells and lymphocytes DON induced up regulation of cytokine expression in jejunum and ileum DON reduced the expression of E-cadherin and OCLN in ileum	[16]
3 mg/kg feed	10 weeks	Growth performance Histomorphometric and immuno-fluorescence investigations of small intestinal epithelium	DON decreased the feed intake (grower) DON increased the crypt depth in jejunum No effect on villus height and ZO-1 expression in jejunum and ileum	[24]
2.29 mg/kg feed	4 weeks	Intestinal morphology/histological scores	DON induced atrophy and villus fusion, necrotic debris and areas of enterocytes lyses DON caused 15% lower histological scores in jejunum	[25]
2.85 mg/kg feed	5 weeks	Claudin-4 (CLDN4) expression (Western blot, immunofluorescence staining) in jejunum	DON reduced CLDN4 expression in jejunum	[26]
2.8 mg/kg feed	4 weeks	Growth performance, intestinal microflora	DON reduced the daily weight gain (first week) Moderate effect on cultivable bacteria in the intestine	[27]
1.2–2 mg/kg feed	84 days	Gene expression in ileum	DON induced a downregulation of interleukin-1 beta (IL-1β) and IL-8 expression in ileum	[28]
12 µg/kg BW/day	42 days	Absorption, accumulation and final presence of DON in the gastrointestinal tract	Presence of DON in intestinal tissues: DON concentrations in small intestine ranged from 7.2 (in the duodenum) to 18.6 ng/g (in the ileum) and in large intestine from 1.8 (in transverse the colon) to 23.0 ng/g (in the cecum)	[29]
1.5 mg/kg feed	28 days	Weight gain, histological changes in medium jejunum, proximal ileum and mesenteric lymph nodes	DON induced a decrease in villus height of jejunum, and a reduction in crypt depth of jejunum and ileum DON induced a decrease in number of mitotic figures, goblet cells in jejunum and ileum DON induced a decrease in number of lymphocytes in jejunum DON induced significant increase in lesional score and caspase-3 positive cells in lymph nodes	[30]

## 2. Results

### 2.1. Average Daily Gain is Decreased by 0.9 ppm DON in the Diet

No alterations were observed in the general health conditions of the piglets during the experimental period. However, the growth performance of piglets fed the DON diet was affected, since these piglets showed a significantly lower relative weight gain, as well as a lower average daily gain (kg/day) compared to control piglets. There was no obvious difference between the total feed intake of the group piglets fed a DON diet compared to the group fed a control diet. However, a higher feed conversion ratio was observed in the group piglets fed the DON diet (Table 2).

**Table 2:** Body weight (BW), relative weight gain, average daily gain, feed intake and feed conversion ratio.

Item	Start weight (kg)		End weight (kg)		Relative weight gain (% increase)		Average daily gain (kg/day)		Feed intake (kg/day)	Feed conversion ratio
Exp. group	Mean	S.E.M.	Mean	S.E.M.	Mean	S.E.M.	Mean	S.E.M.	Mean	Mean
**Control**	8.67	0.48	10.98	0.53	27.21	1.82	0.29	0.01	0.31	1.12
**DON**	7.87	0.47	9.48	0.61	20.17 *	1.15	0.20 ***	0.01	0.30	1.57

** *p*-value <0.01; *** *p*-value <0.001; relative weight gain = ((end weight-start weight)/start weight) **×** 100% per individual animal.

### 2.2. Detectable DON in Plasma Levels after Bolus Administration

The average values of plasma DON in the piglets sampled at 2 h after receiving a DON bolus (0.28 mg/kg BW) and fed a DON diet for 10 days were 168 ± 16.1 ng/mL. DON plasma levels were below the detection limit in the control group (detection limit: 0.05 µg/mL).

### 2.3. Low DON Levels Can Induce Histomorphological Changes in the Piglet Intestine

**Figure 1:**
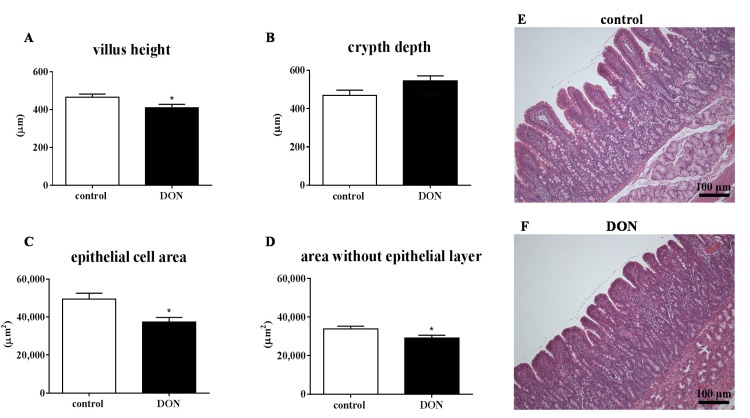
DON induces histomorphological changes in the piglet duodenum even at low exposure levels. Piglets were fed a control or DON diet (0.9 mg/kg feed) for 10 days; two hours after the DON bolus (0.28 mg/kg BW), segments obtained from duodenum were fixed in 10% formalin, and paraffin sections were H&E-stained for histomorphometric analysis of villus height (**A**), crypt depth (**B**), epithelial cell area (**C**) and area without epithelial layer (**D**); representative photomicrographs of villi in duodenum (Figure 1E,F) from control (**E**) and DON-fed piglets (**F**). Magnification: 100×. Results are expressed as means ± SEM; *n* = 9–10 animals/group. * *p* ≤ 0.05; significantly different from the control group.

**Figure 2:**
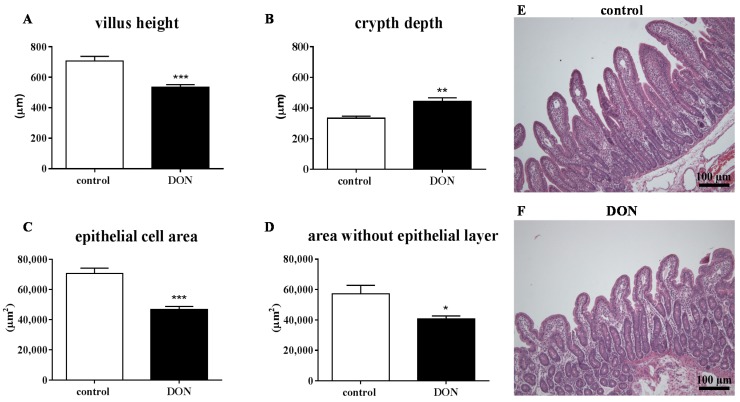
DON induces histomorphological changes in the piglet jejunum even at low exposure levels. Piglets were fed a control or DON diet (0.9 mg/kg feed) for 10 days; two hours after the DON bolus (0.28 mg/kg BW), segments obtained from jejunum were fixed in 10% formalin, and paraffin sections were H&E-stained for histomorphometric analysis of villus height (**A**), crypt depth (**B**), epithelial cell area (**C**) and area without epithelial layer (**D**); representative photomicrographs of villi in jejunum from control (**E**) and DON-fed piglets (**F**). Magnification: 100×. Results are expressed as means ± SEM; *n* = 9–10 animals/group. * *p* < 0.05, ** *p* < 0.01, *** *p* < 0.001; significantly different from the control group.

Providing a DON-contaminated diet resulted in a decreased villus height (Figure 1A and Figure 2A), epithelial cell area (Figure 1C and Figure 2C) and area without epithelial layer (Figure 1D and Figure 2D) in both duodenum and jejunum. The crypt depth was significantly increased in the jejunum of DON-treated piglets compared to piglets fed a control diet (Figure 2B). Representative photomicrographs of villi in duodenum (Figure 1E,F) and jejunum (Figure 2E,F) from control (Figure 1E and Figure 2E) and DON-fed piglets (Figure 1F and Figure 2F) are depicted in Figure 1 and Figure 2 and showed that villi in both duodenum and jejunum of piglets given the DON diet for 10 days were proportionally smaller compared to the villi from control animals. The ratio between the epithelial cell area and villus area without epithelial cell area remained the same in DON-fed animals compared to the control animals Figure S1). Additionally, in the duodenum, a slight, but significant decrease in the villus breadth top is observed in the pigs receiving a DON diet, while in the jejunum, the villus breadth base is significantly decreased in the DON-fed pigs compared to control pigs (Figure S1).

### 2.4. Several Markers for Barrier Integrity, Inflammation and Oxidative Stress in the Intestines Are Affected by the DON Diet

#### 2.4.1. Tight Junction Proteins

**Figure 3:**
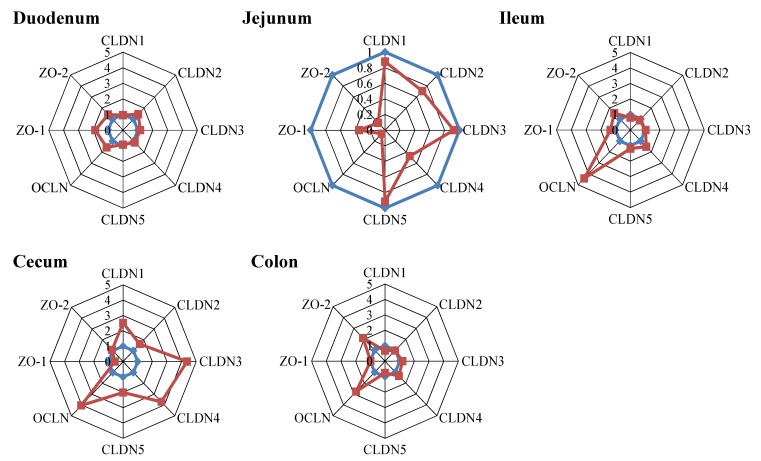
The mRNA expression levels of markers of barrier integrity are affected by short-term, low-dose exposure to DON. Piglets received a control (blue line) or DON diet (0.9 mg/kg feed, red line) for 10 days; two hours after the DON bolus, samples from different parts of the intestine (duodenum, jejunum, Ileum, cecum and colon) were collected, and mRNA levels of various tight junction (TJ) proteins (CLDN1, 2, 3, 4, 5, ZO-1, ZO-1 and OCLN) were measured by RT-PCR. Results are expressed as the relative mRNA expression as means ± SEM; *n* = 9–10 animals/group.

RT-PCR analysis was performed to investigate DON-induced alterations in barrier integrity by measuring the mRNA expression levels of various tight junction (TJ) proteins, including claudin (CLDN) 1-5, occludin (OCLN), zona occludens protein-1 (ZO-1) and zona occludens protein-2 (ZO-2) in different parts of the intestine (duodenum, jejunum, ileum, cecum and colon). The mRNA expression of ZO-1 and OCLN was upregulated in duodenum of piglets fed a DON diet compared to piglets fed a control diet (Figure 3, duodenum). Gene expression levels of CLDN4, OCLN, ZO-1 and ZO-2 in jejunum of DON-treated piglets were clearly downregulated compared to control animals (Figure 3, Jejunum). In the ileum of piglets fed the DON diet, especially the mRNA expression of OCLN was upregulated compared to the control animals (Figure 3, ileum). Increased mRNA expression levels of CLDN1, CLDN3, CLDN4, CLDN5 and OCLN were observed in the cecum of piglets fed a DON diet compared to the control diet (Figure 3, cecum). An increase in mRNA levels of OCLN and ZO-2 related to the DON diet was found in the colon of piglets fed the DON-contaminated diet (Figure 3, colon).

**Figure 4:**
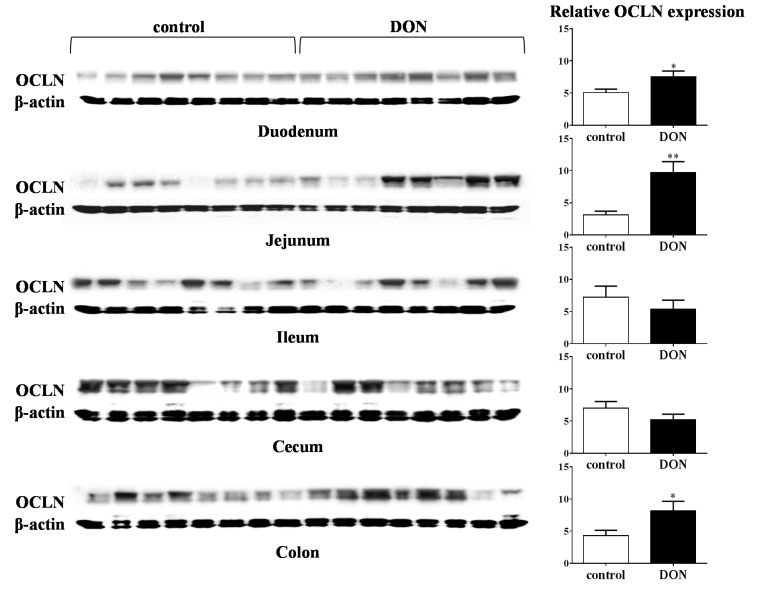
The protein expression of occludin in different parts of the intestine are affected by short-term, low-dose exposure to DON. Piglets received a control diet or DON diet (0.9 mg/kg feed) for 10 days; two hours after the DON bolus, samples from different parts of the intestine (duodenum, jejunum, Ileum, cecum and colon) were collected, and protein levels were measured by Western blot analysis. Results are expressed as the relative protein expression (normalized to β-actin) as means ± SEM; *n* = 8 animals/group. * *p* ≤ 0.05, ** *p* < 0.01; significantly different from the control group.

Additionally, low-dose DON exposure also affected the protein expression of OCLN in the different parts of the intestine. As illustrated in Figure 4, the protein expression of OCLN is significantly increased in duodenum, jejunum and colon of DON-treated animals compared to control animals.

#### 2.4.2. Inflammatory Markers

**Figure 5:**
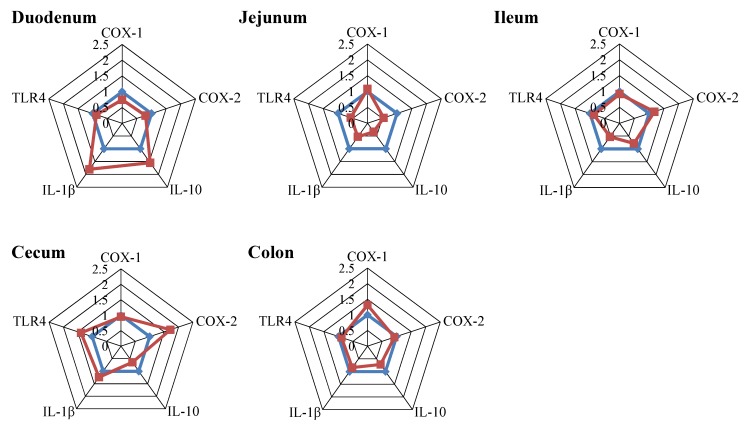
The mRNA expression levels of markers of inflammation are affected by short-term, low-dose exposure to DON. Piglets received a control (blue line) or DON diet (0.9 mg/kg feed, red line) for 10 days; two hours after the DON bolus, samples from different parts of the intestine (duodenum, jejunum, Ileum, cecum and colon) were collected, and mRNA levels of inflammatory markers (COX-1, COX-2, IL-10, IL-1β and TLR4) were measured by RT-PCR. Results are expressed as the relative mRNA expression as means ± SEM; *n* = 9–10 animals/group.

The effect of DON on the development of inflammation was investigated by measuring the mRNA expression levels of cyclooxygenase-1 (COX-1), COX-2, interleukin-1 beta (IL-1β), interleukin-10 (IL-10) and Toll-like receptor 4 (TLR4) in the intestine. IL-10 and IL-1β mRNA levels were increased in the duodenum of piglets fed a DON diet compared to a control diet (Figure 5, duodenum), while they were slightly decreased by DON in the jejunum (Figure 5, jejunum). Enhanced COX-2 mRNA levels were found in cecum of DON-treated piglets compared to control piglets (Figure 5, cecum). COX-1 and TLR4 remained unchanged after DON treatment.

#### 2.4.3. Oxidative Stress Markers

**Figure 6:**
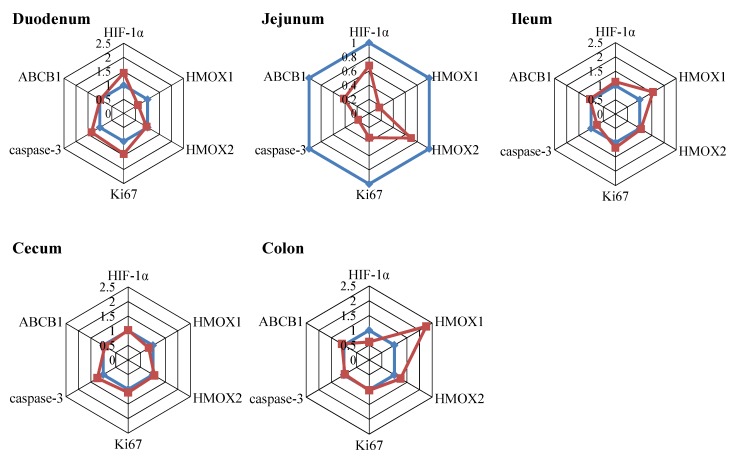
The mRNA expression levels of markers of oxidative stress, efflux transporter, proliferation and apoptosis are affected by short-term, low-dose exposure to DON. Piglets received a control (blue line) or DON diet (0.9 mg/kg feed, red line) for 10 days; two hours after the DON bolus, samples from different parts of the intestine (duodenum, jejunum, Ileum, cecum and colon) were collected, and mRNA levels of stress markers (HIF-1α, HMOX1, HMOX2), an apoptotic and a proliferative marker (caspase-3 and Ki67) and an efflux transporter (ABCB1) were measured by RT-PCR. Results are expressed as the relative mRNA expression as means ± SEM; *n* = 9–10 animals/group.

In addition to the specific markers of inflammation, the mRNA levels of hypoxia-inducible factor 1-alpha (HIF-1α), heme-oxigenase1 (HMOX1) and heme-oxigenase2 (HMOX2) were measured in the intestine as potential markers of oxidative stress. HMOX1 was downregulated in the jejunum after exposure to a DON-contaminated diet (Figure 6, jejunum), while especially in the colon of piglets fed the DON diet, increased HMOX1 levels were identified as compared to piglets fed the control diet (Figure 6, colon).

#### 2.4.4. Efflux Transporter

As DON is a substrate for efflux transporters, the permeability glycoprotein (P-gp, ATP-binding cassette, sub-family B member 1 (ABCB1)) mRNA expression levels were assessed in different parts of the intestine. Downregulated mRNA expression levels of ABCB1 were only detected in the jejunum after DON exposure (Figure 6, jejunum).

#### 2.4.5. Proliferation and Apoptosis

**Figure 7:**
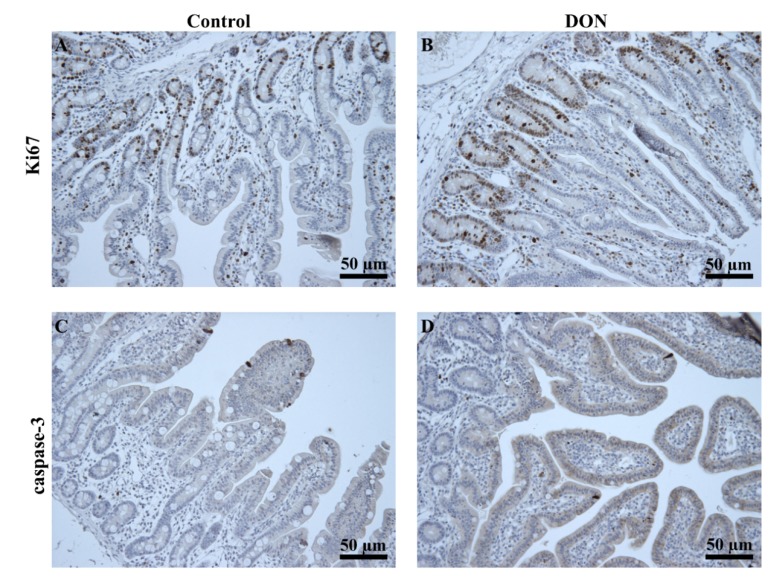
No obvious changes in the expression of proliferation marker Ki67 and apoptotic marker caspase-3 between jejunum of control and DON-treated piglets. For immunohistochemical staining, Swiss-rolled paraffin sections obtained from the jejunum of control (**A**,**C**) and DON-treated (**B**,**D**) piglets were detected by antibodies for Ki67 or caspase-3, as described in the Materials and Methods. Representative images are shown (*n* = 5 piglets/group). Magnification: 200×.

The mRNA levels of Ki67, a proliferative marker, and caspase-3, a marker for apoptosis, were downregulated in jejunum of piglets fed the DON diet compared with piglets fed the control diet (Figure 6, jejunum). No remarkable effects were observed in the other parts of the intestine (Figure 6, duodenum, ileum, cecum and colon). No obvious changes were observed in the immuno-histochemical staining for Ki67 and caspase-3 between the jejunum of piglets fed the DON diet and piglets fed the control diet (Figure 7).

## 3. Discussion

The aim of this study was to identify biomarkers related to gut health that might be indicative for changes at low exposure level, hence supporting risk characterization of DON in pigs and the assessment of intervention strategies. Growing pigs were given low DON levels (0.9 mg/kg feed) in feed for 10 days, which is the recommended maximal concentration at which no signs of clinical intoxications are expected. Conversion of the concentration in feed (*i.e.*, 0.9 ppm) and the daily feed intake per kg/BW, this in-feed application corresponded to a dose varying between 0.04 mg/kg BW at the beginning to 0.03 mg/kg BW at the end of the 10 days exposure period.

### 3.1. Growth and Performance

The most common clinical signs due to prolonged dietary exposure to DON in different experimental animal models are depression in feed intake and weight gain [11,31,32,33]. Different studies reported that pigs could tolerate up to 0.6–0.9 mg of dietary DON/kg feed without any adverse effects on feed consumption and BW gain (European Food Safety Authority, 2004; Table 3) [1]. In the present study, the average (relative) daily weight gain was negatively affected by the given dose of DON, and subsequently, an enhanced feed conversion ratio was calculated for the DON group. The total feed intake of the group piglets fed a low-dose DON diet remained the same as the group piglets fed a control diet, which is also observed in other studies [34,35]. This significant effect on the average daily gain by this low contamination level of DON in diet together with a short exposure period of 10 days was not expected. However, an old study conducted with naturally-contaminated grains showed a temporary reduction on feed intake even in pigs given a low DON dose in the diet (below 1 mg DON/kg feed) [36].

**Table 3:** Primer sequences of genes used for qRT-PCR analysis.

Target gene	Primer sequence (5′-3′)		AT	Reference sequence
	Forward	Reverse		
**ABCB1**	TGGCAGTGGGACAGGTTAGTTC	CACGGTGCTTGAGCTGTCAATC	65	AY825267
**Caspase-3**	AGAGGGGACTGCTGTAGAACT	CCGTCTCAATCCCACAGTCC	58.7	NM_214131.1
**CLDN1**	TGGCTCCGCGTCTCAGTCC	TGCGAGGGGTGCAGGTCTAA	65	NM_001244539.1
**CLDN2**	CTCGTTGGCCTGTATCATCACC	CAGGGGGGAGTAGAAGTCCC	63.1	NM_001161638.1
**CLDN3**	AACACCATCATCCGGGACTTC	CGCGGAGTAGAGGATCTTGG	61.2	NM_001160075.1
**CLDN4**	AGGAGAGACGCTTCAATCGG	GTCCAGACACCTGAACACCG	63.1	NM_001161637.1
**CLDN5**	CTCTGCTGGTTCGCCAACA	CAGCTCGTACTTCTGCGACATG	58.7	NM_001161636.1
**COX-1**	CAAGATGGGTCCTGGCTTCA	CCATAAATGTGGCCGAGGTCTA	64.3	XM_001926129.4
**COX-2**	CATTGATGCCATGGAGCTGTA	CTCCCCAAAGATGGCATCTG	64.3	NM_214321.1
**HIF-1α**	GCTTGCTCATCAGTTGCC	GCCTTCATTTCATCTTCAATATCC	64.3	AY485675.1
**HMOX1**	AGACCGCCTTCCTGCTCA	GGGTCTCTGGTCCTTAGTGTC	64	NM_001004027
**HMOX2**	GCAGCAGTTCAAGCAGTTCT	CCTCCTCCACGATCTTCTCT	63.1	NM_001244412.1
**HPRT**	CTGAACGGCTTGCTCGAGAT	TCCAGCAGGTCAGCAAAGAA	63.1	NM_001032376.2
**IL-10**	CGGCGCTGTCATCAATTTCTG	CCCCTCTTGGAGCTTGCTA	58.7	NM_214041
**IL-1β**	GTGCAAACTCCAGGACAAAGACCA	CACAAGCTCATGCAGAACACCAC	61.2	NM_214055
**Ki67**	TCTTGTCCCTGAATCCGCAA	TGTTTCTCTGGTTGCTTGGTTG	61.2	NM_001101827.1
**OCLN**	ATCAACAAAGGCAACTCT	GCAGCAGCCATGTACTCT	55.8	NM_001163647.2
**TLR4**	CAAGGACCAGAAGCAGCTCC	GACGGCCTCGCTTATCTGAC	63.1	AB188301.2
**ZO-1**	GAGTTTGATAGTGGCGTT	GTGGGAGGATGCTGTTGT	58.7	XM_005659811.1
**ZO-2**	GCAGAGACAACCCCCACTTT	CGTTAACCATGACCACCCGA	55.8	NM_001206404.1

### 3.2. DON in Plasma

In the current study, the average DON levels in plasma of piglets fed a DON diet for 10 days sampled at 2 h after receiving a DON bolus (0.28 mg/kg BW) were 168 ± 16.1 ng/mL. This maximum absorption rate was determined as a reference point for further studies with dietary components that may impair the absorption of DON. In a mouse study, DON levels observed in plasma 120 min following acute oral DON exposure (5 mg/kg BW) were 260 ng/mL [37]. Comparable data in pigs are lacking. However, in chronic exposure studies with DON in pigs, much lower DON levels in plasma were observed, since data by these studies showed DON levels in blood of 5–17 (10.5) ng/mL after a chronic DON diet (2.3 mg/kg feed) for 28 days [38] and 7–30 (22.5) ng/mL after a DON diet (4.5 mg/kg feed) for five weeks [31]. In one of the recently published studies, DON plasma levels were 13.2 ng/mL after a DON diet (3.1 mg/kg feed) for 37 days [39].

### 3.3. Effects of DON on Intestinal Morphology

Intestinal epithelial cells are considered to be one of the primary targets following dietary DON exposure, since DON mainly enters the body via the oral route [40]. The most prominent alterations in the current investigations concerned villus flattening along the intestine with a related decrease in epithelial cell area and the area without epithelial layer (mucosal area) in both duodenum and jejunum of DON-exposed animals, while the intestinal epithelial layer was not disrupted. Alternatively to the assumption that the morphological changes are related to the direct effects of the epithelial cells, Blikslager *et al.* [41] discussed that the decreased villus height could be explained by villus contraction, which aids the restoration of the barrier function via reducing the surface area. This decrease in villus height and corresponding reduced surface area in the intestines may impair nutrient transport and utilization, which could at least partly explain the decrease in average daily gain in the DON-fed animals. The ability of DON to decrease nutrient absorption has been previously reported ([17,42,43] and reviewed in [3]). Various other studies also observed a smaller villus height in jejunum after dietary DON exposure, albeit at higher exposure levels (Bracarense *et al.* [16], DON 3 mg/kg feed for five weeks; Lucioli *et al.* [21], DON 2.3 mg/kg feed for 35 days; Pinton *et al.* [25], DON 2.3 mg/kg feed for four weeks).

In contrast to the decrease in villus height after DON exposure, the crypt depth is increased in the jejunum of piglets exposed to DON in the current study. This is consistent with observations of Dänicke *et al.* [24], who found DON-related effects on crypt depth in the jejunum (3.1 mg DON/kg feed for 10 weeks). It is believed that deeper crypts are an indication of a high cell turnover to permit the renewal of the villus in response to inflammation and toxic damage [44,45]. In light of this assumption, we investigated whether DON exposure indeed affected the proliferation rate of intestinal cells, and subsequently, the proliferative marker Ki67 was evaluated. Downregulated mRNA levels of Ki67 in the jejunum of DON-fed pigs were found, while no obvious changes in proliferating and apoptotic cells were observed in the immunohistochemistry staining for Ki67 and caspase-3 in the jejunum of DON-fed piglets compared to control piglets. This would suggest that at this low level, DON does not clearly influence the cell turnover in the intestines.

### 3.4. Barrier Function

The intestinal barrier, the first target for toxins, like DON, is composed of epithelial cells connected by an intercellular junctional complex consisting of tight junctions, adherens junctions, gap junctions and desmosomes, among which the TJs represent the major determinants of the paracellular permeability. The TJ protein OCLN is an important integral membrane TJ protein involved in TJ stabilization and the formation of a highly effective barrier [46]. Results of the present study show that especially the tight junction protein OCLN was upregulated by DON exposure in different parts of intestine (duodenum, ileum, cecum and colon). This effect, an upregulation of OCLN after DON exposure, is also reported in *in vitro* studies with Caco-2 cells (4.17 µM DON for 3 h, 6 h and 24 h [6], 5000 ng/mL for 24 h [47]). *In vivo* studies related to chronic DON exposure in piglets mainly focus on the effect on TJ protein expression in the intestine without any investigation on gene expression levels [16,26]. Here, it has been illustrated that the protein expression of OCLN in duodenum, jejunum and colon was significantly increased after low-dose DON exposure compared to control pigs, which is in line with the increase in OCLN mRNA expression in duodenum and colon. Surprisingly, there is a discrepancy between the decrease in mRNA expression of OCLN in the jejunum of piglets fed a DON diet and the increase in OCLN protein expression in the jejunum after a DON diet. There is no clear explanation for these findings, but we can speculate that this could be related to the short-term, low-dose exposure to DON. A higher DON dose (3 mg/kg feed) for a longer period (five weeks) resulted in a reduced OCLN protein expression in the pig ileum [16]. An *in vitro* study by Jeong Gu *et al.* [48] also showed a decrease in OCLN protein expression in IPEC-J2 cells after DON exposure (2 µg/mL) for 48 h.

Akbari *et al.* [6] described that in mice, the main effect on the TJ network exerted by a high acute DON dose (25 mg/kg BW) is the modulation of the CLDN expression. In the current experiment, an upregulation of claudins, CLDN-1, -3 and -4, was observed in the cecum of DON-fed piglets, while in the jejunum, the mRNA expression of different TJ proteins (OCLN, ZO-1 and ZO-2) is downregulated. Recently, Lessard *et al.* [49] also showed a down-regulation in mRNA expression of CLDN-3, -4 and OCLN in the ileum of piglets fed a DON diet (3.5 mg /kg feed) for 42 days compared to controls.

The clinical relevance of these various alterations in tight junction protein expression along the intestine cannot be clearly explained, but predict a difference in the TJ expression pattern and the susceptibility of the different parts of the intestine against DON. A comprehensive study in the mice intestine pointed out that the adult mouse intestine expresses many different claudins in complex quantitative and spatial patterns, and this pattern could also be affected by age [50]. Comparable studies in pigs are not available, but would be needed for further interpretations of the obtained results. Another explanation could be the difference in exposure side in different parts of the intestine, since DON absorption mainly occurred in the upper small intestine, while the large intestine is also exposed to DON via the blood stream [51]. This is reinforced by Diesing *et al.* [52], who showed that apical and basolateral exposure of DON to epithelial cell layers trigger different gene response profiles. Akbari *et al.* [6] also observed differences in the routes of DON exposure in an *in vitro* Caco-2 transwell system. Interestingly, Waśkiewicz *et al.* [29] showed that administration of low-dose DON to piglets resulted time-dependently in increasing DON concentrations in the intestines, while the relative DON concentrations in the liver decreased. Within the intestinal tissue, the DON tissue concentrations varied in the different segments, with increasing concentrations over time in the ileum. These time-dependent changes could be a possible explanation for the differences in mRNA expression between the different intestinal segments.

### 3.5. Immune Parameters

A number of studies suggest that DON is a potent immuno-modulator acting as an immunosuppressive, as well as an immuno-stimulatory agent [53]. Most *in vivo* studies focus on a wide array of pro-inflammatory cytokines and chemokines that are detectable in different organs, like spleen, liver, kidney and lung, after DON exposure [54,55,56]. The mRNA expression of different cytokines and chemokines along the intestine after dietary DON is not yet abundantly investigated. In this study, both IL-1β and IL-10 mRNA levels were increased in the duodenum of piglets fed a DON diet compared to control piglets. Bracarense *et al.* [16] showed that in ileum or jejunum of piglets fed a DON diet (3 mg/kg feed) for five weeks the TNF-α, IL-1β, IFN-γ, IL-6 and IL-10 expression was significantly upregulated, while the results of Lessard *et al.* [49] revealed that mRNA expression of IL-8, CXCL10 and IFN-γ was upregulated in ileum of pigs fed a DON-contaminated diet (3.5 mg DON/kg feed). In mice, it has been observed that DON at high doses of 25 mg/kg BW within 2 h transcriptionally upregulates the expression of IL-10 and IL-1β in Peyer’s patches [57], while IL-1β, IL-6 and TNF-α mRNA levels in Peyer’s patches were upregulated after short-term repeated oral exposure to DON (5 mg/kg BW) [54]. In contrast, Becker *et al.* [28] observed a downregulation for IL-1 β and IL-8 in ileum from piglets after chronic exposure to low-level DON and discussed that the decreased expression of the cytokines is due to the indirect DON-mediated effect of reduced feed consumption. We observed a tendency towards downregulated cytokine levels in the jejunum of piglets fed a DON diet.

Furthermore, in the large intestines, where COX-2 is one of the most prominent markers of inflammation, a slight increase in COX-2 mRNA level was observed in the cecum of piglets fed a DON diet. This is comparable to different *in vitro* studies showing also an increase in COX-2 expression in intestinal epithelial cell lines [58,59], as well as in macrophages [53,60,61].

### 3.6. Cellular Oxidative Stress

Cellular oxidative stress is one of the non-specific responses of cells to toxic or inflammatory injury. Oxidative stress can be the source of degradation in membrane phospholipids, which corresponds to a decrease in membrane transport, integrity and to a hyperpermeability of the intestinal barrier [62]. Studies with different cell lines indicated that DON has a capacity to induce oxidative stress [58,63,64]. One of the most sensitive indicators of oxidative stress is HMOX1, and in this study, the mRNA levels of HMOX1 were upregulated in the colon of the DON-fed piglets. Another recent piglet study described an effect of DON on other oxidative stress markers, since an increase in glutathione peroxidase 2 was observed, whereas expression of genes encoding enzymatic antioxidants and superoxide dismutase 3 were downregulated in pigs fed a DON diet (3.5 mg/kg feed) for 42 days [49]. Sheth *et al.* [65] and Basuroy *et al.* [66] described an effect of oxidative stress on the phosphorylation and redistribution of OCLN and ZO-1 in the TJ complex of the intestinal barrier, which could partly explain the observed changes in OCLN expression along the intestine.

### 3.7. Efflux Transporters

Different studies reported the fact that DON is a substrate for ABC transporters influencing the rate of absorption in the upper part of intestine and facilitating secretion of circulating DON in lower parts [67,68]. It should be stressed that in our study, the low doses of DON did not result in an alteration in ABCB1 mRNA levels in different parts of intestine, except a downregulation in the jejunum, which is in line with a previous study where DON (1 and 3 ppm) decreases intestinal ABCB1 expression within 10 days [69].

In our study, a low dose of DON was added to the DON diet, and at the end of the experiment, the animals received a DON bolus. It cannot entirely be excluded that the bolus application influenced some of the results, particularly regarding the PCR results in the upper intestines. However, almost no changes in mRNA expression in the duodenum were observed, and only small differences in the histomorphology between duodenum and jejunum were detected.

## 4. Materials and Methods

### 4.1. Animals

This study was conducted with twenty 4-week-old piglets (Dutch Landrace) obtained from a commercial pig farm (weaning age: 3 weeks old). Animals were housed under conventional conditions at the experimental farm for pig production, Test and Training Center for Agriculture (Proef-en Vormingscentrum voor de Landbouw; PVL) in Bocholt, Belgium, and temperature and lightening programs were followed according to standard recommendations. The piglets were acclimatized for 2 weeks in the experimental facility and allocated to two groups (10 piglets per group) with an equal distribution of sex (4 male/6 female). The groups were housed in separate pens (10 piglets per pen) with free access to feed and water. Piglets were handled according to the Federation of European Laboratory Animal Science Associations (FELASA) guidelines and the Belgian law on the protection of animals, and the experimental protocol was agreed upon by the Ethical Committee on the use of experimental animals.

### 4.2. Experimental Design

Pigs (6 weeks old) were given a commercial standard diet for piglets (Cibus NV, Poperinge, Belgium, Table S1), to which DON was added. DON was incorporated into the diet at 0.9 mg/kg, added as the purified toxin (Romer Laboratories, Inc., Union, MO, USA).

The DON content of the diet used in our experiment was analyzed by HPLC analyses, and no DON contamination (detection limit: 10 µg/kg) was observed in the feed samples of the control diet.

The control and contaminated diets were fed for 10 days. The BW of the piglets was recorded at the start and the end of the experiment, and the individual daily weight gain of the piglets was calculated based on the start and end weights for each individual animal. The start weights of the piglets were not significantly different (Table 2). Additionally, the relative weight gain was calculated by ((end weight-start weight)/start weight) × 100% per individual animal. The amount of feed consumed per group was determined, and the feed conversion ratio was calculated based on the feed intake measured per pen at the beginning and end of the trial. At the final day of the experiment, the animals received a single DON bolus via bottle feeding at a dose of 0.28 mg/kg BW (approximately 10-times higher than the daily DON dose in mg/kg BW) to evaluate the degree of oral absorption. Exactly two hours after the DON bolus, the piglets were sedated with an intramuscular injection of azaperone (Stresnil^®^ Elanco Animal Health, Greenfield, IN, USA) (4 mg/kg) followed by induction of euthanasia via an intravenous injection of 200 mg/kg pentobarbital (Euthasol^®^, Virbac Animal Health, Carros Cedex, France). The entire gastrointestinal tract was removed, and after cutting the mesentery, the small and large intestine were aligned on a table. The small intestine was divided into three parts of equal length, and 10-cm tissue segments were collected from the middle of each part, 10-cm segments collected from the middle part of the cecum and a 10-cm segment of proximal colon collected 30 cm distal to the ileocecal valve [70]. Digesta was removed from these segments by flushing with cold saline.

### 4.3. DON Levels in Plasma

DON levels in blood plasma were measured by standard high-performance liquid chromatography (HPLC) analyses with affinity column cleanup based on the method described in Janes and Schuster [71].

### 4.4. Histomorphometric Analysis of the Small Intestine

The small intestine parts (duodenum and jejunum) were fixed in 10% neutral buffered formalin, embedded in paraffin, and 5-µm sections were cut and stained with hematoxylin and eosin (H&E), according to standard methods. Photomicrographs were taken with an Olympus BX50 microscope (Olympus Europa GmbH, Hamburg, Germany) equipped with a Leica DFC 320 digital camera (Leica Microsystems, Wetzlar, Germany). The morphometric analysis of the sections was performed on 10 randomly-selected, well-oriented villi and crypts per animal. A computerized microscope-based image analyzer (Cell^D version 3.3; Olympus Europa GmbH, Hamburg, Germany, 2009) was used to determine histomorphometric parameters: villus height (measured from the tip of the villus to the villus-crypt junction), crypt depth (measured from the crypt villus junction to the base of the crypt), villus surface area (total surface of the villus), epithelial cell area (villus surface area minus villus area without epithelial cells), villus width, villus breadth top and villus breadth base. These regions of interest were manually defined for each villus separately.

### 4.5. Determination of mRNA Expression in Intestinal Samples by qRT-PCR

For mRNA studies, the intestinal tissue segments (approximately 2–3 cm) were snap frozen in liquid nitrogen and stored at −80 °C for RNA isolation. Fifty milligrams of each sample were suspended in 350 μL RNA Lysis Buffer with β-mercaptoethanol and homogenized using a TissueLyser (Qiagen, Hilden, Germany) for 1 min/25 Hz. RNA isolation was performed using spin columns according to the manufacturer’s instructions (Promega, Madison, WI, USA). Subsequently, 1 µg of extracted total RNA was reverse transcribed with the iScriptTM cDNA Synthesis kit (Bio-Rad Laboratories, Hercules, CA, USA). After reverse transcription, qRT-PCR was performed using a reaction mixture, containing 10 µL of the diluted cDNA mixed with 12.5 µL iQSYBR Green Supermix (Bio-Rad Laboratories, Hercules, CA, USA), forward and reverse primers (final concentration of 300 nM for each primer) and sterile deionized water, prepared according to the manufacturer’s instructions. The MyIQ single-color real-time PCR detection system (Bio-Rad Laboratories, Hercules, CA, USA) was used with MyIQ System 1.0.410 software (Bio-Rad Laboratories, Hercules, CA, USA, 2008). Gene specific primers for CLDN1, CLDN2, CLDN3, CLDN4, CLDN5, OCLN, ZO-1, ZO-2, caspase-3, Ki67, ABCB1, HIF-1α, HMOX1, HMOX2, COX-1, COX-2, IL-1β, IL-10 and TLR4 (Table 3) were derived from the NCBI GenBank and were manufactured commercially (Eurogentec, Seraing, Belgium). The specificity and efficiency of selected primers (Table 3) were confirmed by qRT-PCR analysis of dilution series of pooled cDNA at a temperature gradient (55 °C–65 °C) for primer-annealing and subsequent melting curve analysis. Hypoxanthine phosphoribosyltransferase 1 (HPRT1) was used as reference gene, since HPRT1 is a valid reference gene for transcripts in different pig tissues [72].

### 4.6. Western Blot Analysis

Approximately 50 mg of each intestinal sample were lysed using 500 µL RIPA lysis buffer (Thermo scientific, Rockford, IL, USA) containing protease inhibitors (Roche Applied Science, Penzberg, Germany), and the total protein concentration was measured by the BCA protein assay kit (Thermo scientific, Rockford, IL, USA). Standardized protein amounts of boiled samples were isolated by electrophoresis (CriterionTM Gel, 4%–20% Tris-HCL, Bio-Rad Laboratories, Hercules, CA, USA) and electro-transferred onto polyvinylidene difluoride membranes (Bio-Rad, Veenendaal, The Netherlands). Membranes were blocked with PBS supplemented with 0.05% Tween-20 (PBST) and 5% milk proteins and incubated overnight at 4 °C with antibodies for anti-occludin (1:250, Abcam, Cambridge, UK). After washing in PBST, the membranes were incubated with an appropriate horseradish peroxidase-conjugated secondary antibody (1:5000, Dako, Glostrup, Denmark) for 2 h at room temperature. Finally, blots were washed in PBST, incubated with ECL Prime Western Blotting Detection Reagent (Amersham Biosciences, Roosendaal, The Netherlands), and digital images were obtained with the ChemiDoc MP imager (Bio-Rad Laboratories, Hercules, CA, USA). In the next step the membranes re-probed with a β-Actin antibody (1:4000, Cell Signaling, MA, USA) to assess the equality of loading. Signal intensities were quantified using the ImageJ 1.47 software (National institutes of Health, Bethesda, MD, USA, 2013), and the protein expression was normalized with β-Actin and expressed as the mean fold change in relation to the control group.

### 4.7. Immunohistochemistry

After fixation in 10% formalin, jejunum samples were embedded in paraffin. Paraffin sections (5 µm) were deparaffinized, and endogenous peroxidase activity was blocked with 0.3% H_2_O_2_ (Merck, Darmstadt, Germany) in methanol (30 min) and rehydrated in a graded ethanol series to PBS. After antigen retrieval in 10 mM citrate buffer (PH 6.0) for 10 min in a microwave, sections were pre-treated with 5% goat serum (Dako, Glostrup, Denmark) before overnight incubation with rabbit-polyclonal Ki67 antibody (1:1000, ab66155, Abcam, Cambridge, UK) or caspase-3 (1:1000, Cell Signaling, Danvers, MA, USA) at 4 °C. Tissue sections were sequentially incubated with biotinylated goat-anti rabbit (1:200, Dako, Glostrup, Denmark) followed by streptavidin-biotin complex/horseradish peroxidase (Vectastain Elite ABC, Vector Laboratories, Peterborough, UK). Staining was visualized using 0.05% diaminobenzidine (DAB) solution for 10 min, and sections were counterstained with Mayers’ hematoxylin (Merck Millipore, Amsterdam, The Netherlands). Photomicrographs were taken with an Olympus BX50 microscope equipped with a Leica 320 digital camera.

### 4.8. Statistical Analyses

Experimental results are expressed as the mean ± SEM. Data were analyzed with univariate analysis of variance (ANOVA) using SPSS Statistics 22.0 software (SPSS Inc., Chicago, IL, USA, 2013). Analysis was performed on data in a 2 × 2 (diet × sex) between-subject factorial design. Results were considered statistically significant when *p* < 0.05. A gender effect was only observed in the relative occludin expression in cecum (*p* = 0.015). Differences in feed intake and food conversion ratio between groups were not statistically determined, since these data are based on total feed intake per experimental group.

**Figure 8:**
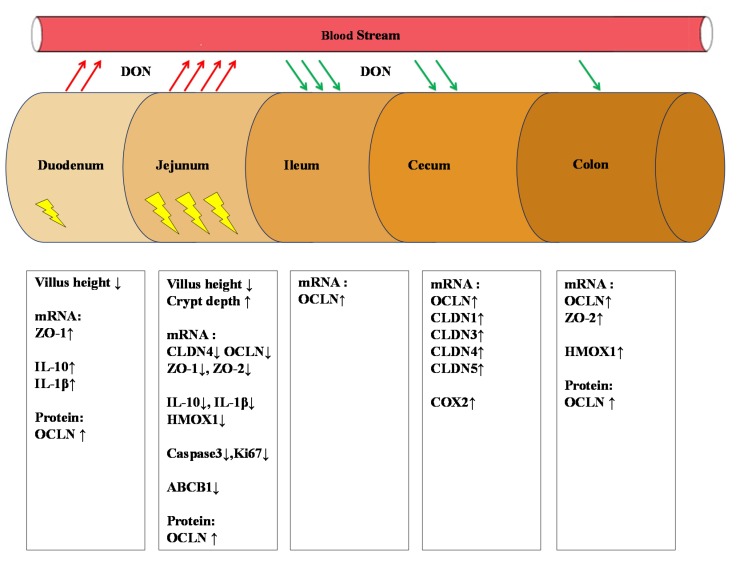
chematic overview of DON-related effects in different segments of the intestine. Red arrows indicate absorption of DON. Green arrows indicate secretion of DON. The rectangles below each segment of the intestine summarize the impact of DON on villus architecture (duodenum and jejunum) and on mRNA expression levels of different genes related to intestinal barrier function (and OCLN protein expression), oxidative stress and inflammation. Negative effects of DON (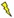) are mainly observed in the jejunum (downregulation of different genes). Up arrows indicate increase and down arrows indicate decrease.

## 5. Conclusions

Taken together, the presented results show that low-level DON exposure (0.9 mg/kg feed) can even, after a short exposure period, impair weight gain accompanied by distinct changes in the intestinal tract of growing piglets, as depicted in Figure 8. Moreover, several indicators associated with intestinal barrier function, oxidative stress and inflammation, identified in previous studies as typical signs of long-term and high-dose exposure to DON, were already altered after the chosen short exposure period of 10 days. These results confirm that the intestines are an important target of DON toxicity, particularly at low dietary doses. This is not only of relevance for a further risk characterization of DON exposure to growing pigs, but might also provide the template for studies devoted to assessment of intervention strategies to mitigate adverse health effects of DON.

## Supplementary Materials

Supplementary materials can be accessed at: http://www.mdpi.com/2072-6651/7/6/2071/s1.
