# Supplementary material for: Deoxynivalenol Impairs Weight Gain and Affects Markers of Gut Health after Low-Dose, Short-Term Exposure of Growing Pigs
Source: Toxins (Basel). 2015 Jun 9;7(6):2071–95. doi: 10.3390/toxins7062071 (PMC4488690; doi:10.3390/toxins7062071)
Supplement: Supplementary file 1 [file toxins-07-02071-s001.pdf]

Supplementary Information

Table S1. Feed composition of standard piglet diet.

Ingredients	Value (g/kg)	Ingredients	Value (g/kg)
Wheat 2012	299.7	Wheat gluten (Roquette, Lestrem, France)	30
Barley 2012	250	Beet pulp sugar	20
Maize 2012	114	Fat + 88% triglyceride	8.5
Soybean (Danex, Izegem, Belgium)	110	Ultracid (INVE, Dendermonde, Belgium)	5
Soya 49/3.5	92.7	Threonine	0.1
Premix (Vimix, Deerlijk, Belgium)	70		
**Nutrients**	**Value**	**Nutrients**	**Value**
Crude protein (g)	171.30	6-phytase (FYT)	2,100.00
Lysine (g)	12.17	Sodium (g)	1.47
Digestible lysine (g)	10.57	Chlorine (g)	3.10
Methionine (g)	4.39	Potassium (g)	7.83
Digestible methionine (g)	3.96	Sodium + potassium-chloride (meq)	192.13
Digestible cystine (g)	2.24	Magnesium (g)	1.57
Digestible methionine + cystine (g)	6.26	Vitamin A per kg (IE)	15,050.00
Digestible threonine (g)	6.55	Vitamin D3 per kg (IE)	2,030.00
Digestible tryptophan (g)	2.00	Vitamin E per kg (IE)	120.33
Sugar + starch (g)	454.83	Butylated hydroxytoluene (ppm)	150.15
Sugar (g)	35.60	Formic acid (ppm)	1,633.00
Crude starch (g)	401.50	Lactic acid (ppm)	2,105.00
Crude fiber (g)	35.96	Moisture (g)	115.86
Net energy (kCal)	2,347.17	Weight (%)	100.00
Crude fat (g)	47.98	Cu premix copper sulfate (ppm)	148.03
Linoleic acid (g)	18.98	Fe premix sulfate (ppm)	198.17
n-3 poly unsaturated fatty acid (g)	2.26	Mn premix (oxide) (ppm)	52.22
n-6 poly unsaturated fatty acid (g)	18.70	Zn premix (sulfate) (ppm)	101.85
Crude ash (g)	49.81	I premix (calcium iodate) (ppm)	1.12
Ca (g)	7.09	Co carbonate (ppm)	1.12
Phosphorus (g)	5.12	Se sodium selenite (ppm)	0.29
Digestible phosphorus pellet (g)	3.50	Bacterial endo-xylanase (IU)	11.06
Digestible phosphorus flour (g)	3.78		
**Nutrient ratio**	**Value**	**Nutrient ratio**	**Value**
Calcium (g)/Phosphorus (g)	1.38	Digestible methionine (g)/ Digestible lysine (g)	0.37
Calcium (g)/Digestible phosphorus flour (g)	1.87	Digestible threonine (g)/ Digestible lysine (g)	0.62
n-6 poly unsaturated fatty acid (g)/ n-3 poly unsaturated fatty acid	8.28	Digestible tryptophan (g)/ Digestible lysine (g)	0.19
Digestible methionine+cystine (g)/ Digestible lysine (g)	0.59		

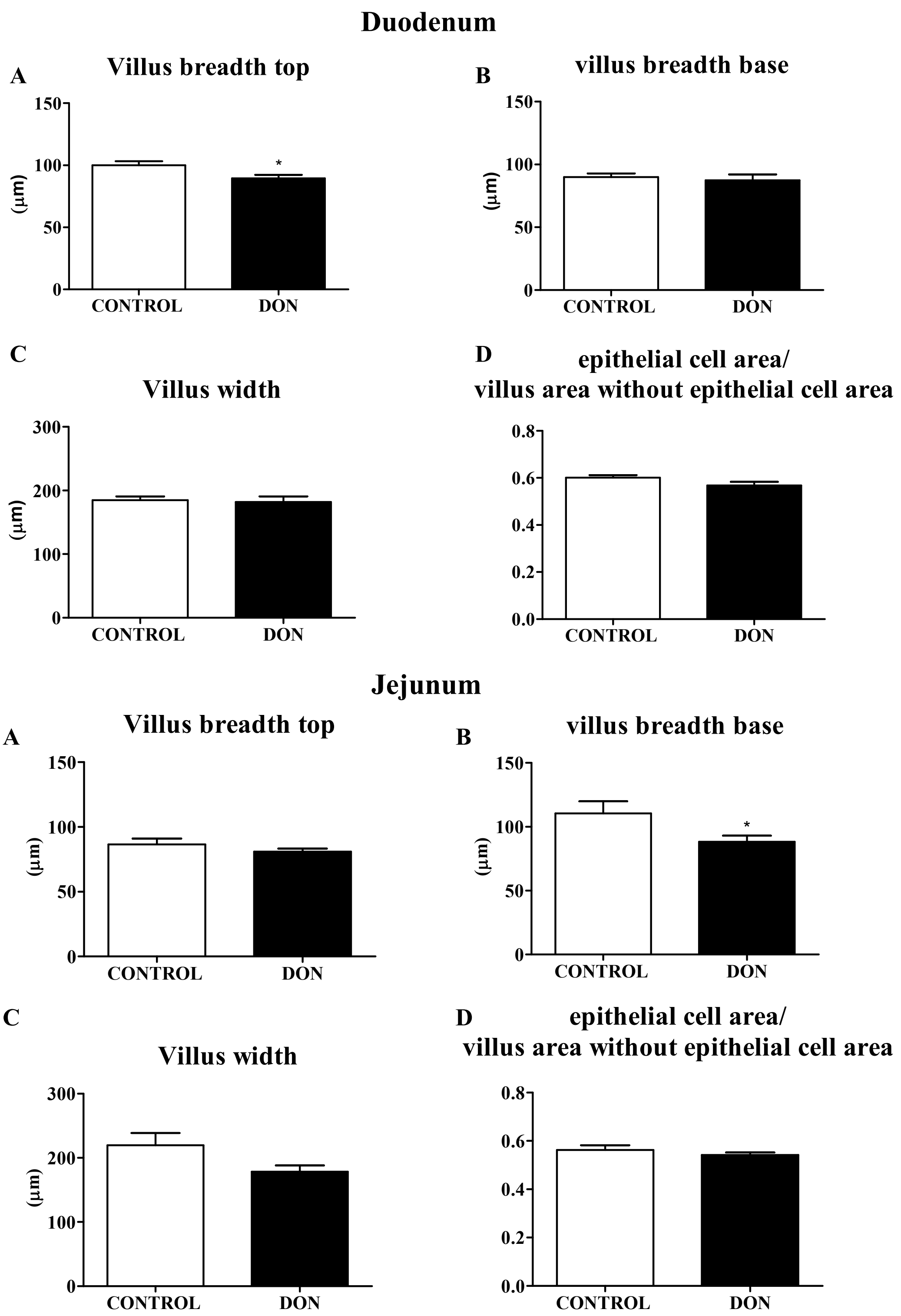

Figure S1. Histomorphological measurements in the piglet duodenum and jejunum. * $p \leq 0.05$; significantly different from the control group.
